# 

**Published:** 1883-12

**Authors:** 


### INDEX.

Abdominal section in perforating-ulcer of stomach—N. C. Dobson, 196.
Abortion, The treatment of neglected—Dr. V. Idelson (*Extract*), 264.
Adonis vernalis (*Extract*), 266.
Alcoholic inebriety from a medical standpoint —Dr. Joseph Parrish (*Review*), 248.
Alexander, Dr.—New method of treating displacements of the uterus (*Extract*), 140.
Aneurism, A case of—Dr. H. Waldo, 232.
Aneurisms, The symptomatology of aortic—Dr. J. Graham Brown (*Extract*), 257.
Antrum, Suppuration in the—W. H. Harsant, 93.
Aorta during parturition, Rupture of the (*Extract*), 139.
Arachnitis (sub-), Purulent — Dr. George Thompson, 107.
Auscultation and percussion—Dr. Samuel Gee (*Review*), 134.

Bandaging, The essentials of—Berkeley Hill (*Review*), 133.
Banks, Mitchell—Free removal of mammary cancer (*Extract*), 137.
Barbour, Dr. A. H. F.—The prophylaxis of ophthalmia neonatorum (*Extract*), 262.
Barnes, Dr. Fancourt—A manual of midwifery for midwives (*Review*), 252.
Beddoe, Dr. J.—Address to Bristol medical school, 235.
Bernard, Charles—The way of cutting for the stone in the kidneys (*Extract*), 136.
Bert, M. Paul—Disease germs; their vitality; means of rendering them innoxious (*Extract*), 256.
Boucheron, Dr. — Cure of squint without operation (*Extract*), 139.
Boys, A. H.—A case of priapism, 224.
Brown, Dr. J. Graham—The symptomatology of aortic aneurisms (*Extract*), 257.

Cancer, Clinical notes on—Dr. H. L. Snow (*Review*), 251.
Cancer, Free removal of mammary—Mitchell Banks (*Extract*), 137.
Cancer, Resection of the pylorus for (*Extract*), 139.
Cardiograph in medicine, The—G. Munro Smith, 71.
Chancre on the lip—W. H. Harsant, 97.
Cholera, Asiatic—C. M. Jessop (*Review*), 252.
Congenital deformity of right leg and left hand—J. Greig Smith, 130.
Congenital syphilis—W. H. Harsant, 83.
Congenital talipes—W. H. Harsant, 97.
Contagion, The proofs of the existence of a phthisical—Dr. R. Shingleton Smith, 1.
Contagiousness of phthisis, Clinical evidence against the—Dr. E. Markham Skerritt, 48.
Convulsions treated by venesection, Puerperal —J. Fuller, 111.
Cooper, Berkeley Hill and Arthur — The student's manual of venereal diseases (*Review*), 134.
Corneal and conjunctival diseases, Iodoform in—Dr. A. G. Heyl (*Extract*), 263.

Deformity of right leg and left hand—J. Greig Smith, 130.
Displacements of the uterus, New method of treating—Dr. Alexander (*Extract*), 140.
Dobson, N. C.—Abdominal section in perforating-ulcer of stomach, 196.
Duncan, Dr. D. P.—Worms (*Extract*), 255.

Embolisms of various organs, Infarctions and —Prof. D. J. Hamilton (*Extract*), 253.
Endocarditis of the tricuspid valve, Ulcerative—Dr. J. E. Shaw, 114.
Ether douche or lavement for local pain, The —Dr. C. H. Hughes (*Extract*), 266.
Evans, Dr. J. Fenton—Cystic neuroma, 109; cases of muscular atrophy and degeneration, 212.

Fæces, Gas-light from (*Extract*), 138.
Farquharson, Dr. R.—A guide to therapeutics (*Review*) 135.
Ferguson, Dr.—Two cases of rupture of the heart (*Extract*), 256.
Fœtus remaining fifty-six years without change in a case of extra-uterine pregnancy—M. Sappey (*Extract*), 263.
Fox, Dr. E. Long—Compression of the spinal cord by sarcomatous growths from the soft membranes, 100.
Fuller, J.—Puerperal convulsions treated by venesection, 111.
Fyffe, Dr. W. J.—Malarial poisoning, 143.

Gall-bladder, Removal of—Dr. Langenbuch (*Extract*), 138.
Gas-light from fæces (*Extract*), 138.
Gastrostomy for malignant stricture of the œsophagus—J. Greig Smith, 122.
Gee, Dr. Samuel—Auscultation and percussion (*Review*), 134.
Germs, Disease; their vitality; means of rendering them innoxious—Dr. Paul Bert (*Extract*), 256.
Gouty inflammation of the testis—F. K. Green, 227.
Green, F. K.—Two cases; one of gouty inflammation of the testis, and another the testicle of mumps, 227.

Hamilton, Prof. D. J.—Infarctions and embolisms of various organs (*Extract*), 253.
Hare, Dr. C. J.—Good remedies out of fashion (*Review*), 247.
Harley, Dr. George—Diseases of the liver (*Review*), 131.
Harsant, W. H.—Surgical out-patient notes, 82; congenital syphilis, 83; lupus vulgaris, 88; strumous synovitis of elbow, 91; suppuration in the antrum, 93; misplaced testicles, 96; chancre on the lip, 97; congenital talipes, 97.
Heart, Two cases of rupture of the—Dr. Ferguson (*Extract*), 256.
Heyl, Dr. A. G.—Iodoform in corneal and conjunctival diseases (*Extract*), 263.
Hill and Arthur Cooper, Berkeley—The student's manual of venereal diseases (*Review*), 134.
Hill, Berkeley—The essentials of bandaging (*Review*), 134.
Hughes, Dr. C. H.—The ether douche or lavement for local pain (*Extract*), 266.

Idelson, Dr. V.—The treatment of neglected abortion (*Extract*), 264.
Infarctions and embolisms of various organs—Prof. D. J. Hamilton (*Extract*), 253.
Iodoform in corneal and conjunctival diseases—Dr. A. G. Heyl (*Extract*), 263.
Iodoform in the treatment of phthisis, Note on the utility of—Dr. R. Shingleton Smith, 218.

Jessop, C. M.—Asiatic cholera (*Review*), 252.

Kidneys, The diagnosis of movable (*Extract*), 254.
Kidneys, The way of cutting for the stone in the—Charles Bernard (*Extract*), 136.

Landesberg, Dr. M.—Stretching the optic nerve (*Extract*), 261.
Langenbuch, Dr.—Removal of gall-bladder (*Extract*), 138.
Levis, Dr. R. J.—Surgical expedients in emergencies (*Extract*), 260.
Liver, Diseases of the—Dr. George Harley (*Review*), 131.
Loreta, Professor—Surgical dilation of the pylorus (*Extract*), 139
Lupus vulgaris—W. H. Harsant, 88.

Malarial poisoning—Dr. W. J. Fyffe, 143.
Mammary cancer, Free removal of—Mitchell Banks (*Extract*), 137.
Medical school, Bristol—Address by Dr. J. Beddoe, 235; prize list, 245.
Meningocele, Case of very large—J. Greig Smith, 129.
Midwifery for midwives, A manual of—Dr. Fancourt Barnes (*Review*), 252.
Mumps, The testicle of—F. K. Green, 229.
Muscular atrophy and degeneration, Cases of—Dr. J. F. Evans, 212.
Myers, Dr. A.—A tape-worm trap (*Extract*), 138.

Nephro-lithotomy, The first (?)—Charles Bernard (*Extract*), 136.
Neuroma, Cystic—Dr. J. Fenton Evans, 109.

Œsophagus, Gastrostomy for malignant stricture of the—J. Greig Smith, 122.
Ophthalmia neonatorum, The prophylaxis of—Dr. A. H F. Barbour (*Extract*), 262.
Optic nerve, Stretching the—Dr. M. Landesberg (*Extract*), 261.
Out-patient notes, Surgical—W. H. Harsant, 82.

Parrish, Dr. Joseph—Alcoholic inebriety from a medical standpoint (*Review*), 248.
Parturition, Rupture of the aorta during (*Extract*), 139.
Pathology, Practical: a manual for students and practitioners—Dr. G. S. Woodhead (*Review*), 250
Phthisical contagion, The proofs of the existence of a—Dr. R. Shingleton Smith, 1.
Phthisis and its laryngeal complications, The pathology of—Dr. Seiler (*Extract*), 258.
Phthisis, Clinical evidence against the contagiousness of—Dr. E. Markham Skerritt, 48.
Pia mater, Minute hæmorrhages in the—Dr. T. S. Sheldon, 225.
Placenta prævia, A record of twenty-one cases of—Dr. J. G. Swayne, 166.
Posture, A study of medical—Dr. J. K. Spender, 184.
Priapism, A case of—A. H. Boys, 224.
Prize list, Bristol medical school, 245.
Prowse, Dr. A. B.—Retinoscopy, 200.
Puerperal convulsions treated by venesection—J. Fuller, 111.
Pylorus for cancer, Resection of the (*Extract*), 139.
Pylorus, Surgical dilation of the—Professor Loreta (*Extract*), 139.

Remedies out of fashion, Good—Dr. C. J. Hare (*Review*), 247.
Retinoscopy—Dr. A. B. Prowse, 200.

Sappey, M.—A fœtus remaining fifty-six years without change in a case of extra-uterine pregnancy (*Extract*), 263.
Sarcomatous growths from the soft membranes, Compression of the spinal cord by —Dr. E. Long Fox, 100
Scarlet fever and cerebro-spinal mening*itis* in horses (*Extract*), 139.
Scirrhus of the upper jaw in a child two years old, Primary—J. Greig Smith, 128.
Seiler, Dr.—The pathology of phthisis and its laryngeal complications (*Extract*), 258.
Shaw, Dr. J. E.—Ulcerative endocarditis of the tricuspid valve, 114.
Sheen, Dr. A.—An epidemic of tetanus, 173.
Sheldon, Dr. T. S.—Minute hæmorrhages in the pia mater, 225.
Skerritt, Dr. E. Markham—Clinical evidence against the contagiousness of phthisis, 48.
Smith, G. Munro—The cardiograph in medicine, 71.
Smith, J. Greig—Gastrostomy for malignant stricture of the œsophagus, 122; recurrent pendulous fatty tumour of thigh, 126; primary scirrhus of the upper jaw in a child two years old, 128; case of very large meningocele, 129; congenital deformity of right leg and left hand, 130.
Smith, Dr. R. Shingleton—The proofs of the existence of a phthisical contagion, 1; note on the utility of iodoform in the treatment of phthisis, 218.
Snow, Dr. H. L.—Clinical notes on cancer (*Review*), 251.
Spender, Dr. J. K.—A study of medical posture, 184.
Spinal cord by sarcomatous growths from the soft membranes, Compression of the—Dr. E. Long Fox, 100.
Squint without operation, Cure of—Dr. Boucheron (*Extract*), 129.
Stomach, Abdominal section in perforating-ulcer of—N. C. Dobson, 196.
Stone in the kidneys, The way of cutting for the—Charles Bernard (*Extract*), 136.
Stricture of the œsophagus, Gastrostomy for malignant—J. Greig Smith, 122.
Strumous synovitis of elbow—W. H. Harsant, 91.
Surgical expedients in emergencies—Dr. R. J. Levis (*Extract*), 260.
Surgical out-patient notes—W. H. Harsant, 82.
Swayne, Dr. J. G.—A record of twenty-one cases of placenta prævia, 166.
Synovitis of elbow, Strumous—W. H. Harsant, 91.
Syphilis, Congenital—W. H. Harsant, 83.

Talipes, Congenital—W. H. Harsant, 97.
Tape-worm trap, A—Dr. A. Myers (*Extract*), 138.
Testicle of mumps, The—F. K. Green, 229.
Testicles, Misplaced—W. H. Harsant, 96.
Testis, Gouty inflammation of the—F. K. Green, 227.
Tetanus, An epidemic of—Dr. A. Sheen, 173.
Therapeutics, A guide to—Dr. R. Farquharson (*Review*), 135.
Thompson, Dr. George—Purulent sub-arachnitis, 107.
Tumour of thigh, Recurrent pendulous fatty —J. Greig Smith, 126.

Ulcerative endocarditis of the tricuspid valve —Dr. J. E. Shaw, 114.
Uterus, New method of treating displacements of the—Dr. Alexander (*Extract*), 140.

Venereal diseases, The student's manual of—Berkeley Hill and Arthur Cooper (*Review*), 134.

Waldo, Dr. H.—A case of aneurism, 232.
Woodhead, Dr. G. S.—Practical pathology: a manual for students and practitioners (*Review*), 250.
Worms—Dr. D. P. Duncan (*Extract*), 255.

J. W. ARROWSMITH, PRINTER, QUAY STREET, BRISTOL.

### Bristol
### Medico-Chirurgical Journal.

---

*Extracts from Notices of No.* 1.

"The Editor must be congratulated upon having produced so readable a number for his first issue. The type and illustrations are also to be commended, and we trust that these efforts will meet with the success they deserve."—*Lancet.*

"It contains a large amount of original matter, is well illustrated."—*Medical Record* (New York).

"The book is nicely got up as regards type and paper, and we are glad to notice that a goodly number of practitioners in the neighbourhood have become subscribers. The *Bristol Medico-Chirurgical Journal* has made an excellent start under favourable auspices."—*Medical Times and Gazette.*

"It also contains numerous clinical records, many of which are of a very interesting character."—*Liverpool Medico-Chirurgical Journal.*

"The clinical cases are especially good, and form a strong feature of the number." —*Birmingham Medical Review.*

"The present number is so full of interesting matter that we look forward with pleasure to the appearance of the second."—*Edinburgh Medical Journal.*

---

In future the *Journal* is to be issued quarterly (March, June, September, December.) Each number will consist of 72 pages, thus making a larger volume than the present one.

The subscription (if paid in advance) will remain at 5/-. The *Journal* will then be delivered *post-free.* A single number will be 1/6.

☞ A Case for Binding the *Journal* in yearly volumes is in preparation, and will shortly be ready for delivery. Price for binding (including case), 1/3 per vol. Case only, 6*d.* each; postage of case, 1*d.*

Orders for cases direct to J. W. Arrowsmith, Quay Street, Bristol.

### Bristol Medico-Chirurgical Society.

### President.

W. JOHNSTONE FYFFE, M.D. Dub.

### President-Elect.

GEORGE F. BURDER, M.D. Aberd.

### Honorary Secretary.

R. SHINGLETON SMITH, M.D., B.Sc. Lond.

### Committee.

*Past Presidents.*

- R. W. COE.

- AUGUSTIN PRICHARD, M.D. Berlin.

- W. MICHELL CLARKE.

- J. G. SWAYNE, M.D. Lond.

- E. LONG FOX, M.D. Oxon.

- JAMES G. DAVEY, M.D. St. And.

NELSON C. DOBSON.
L. M. GRIFFITHS.
F. POOLE LANSDOWN.
E. MARKHAM SKERRITT, M.D., B.S., B.A. Lond.
J. GREIG SMITH, M.B., C.M., M.A. Aberd., F.R.S.E.
W. H. SPENCER, M.D., M.A. Cantab.

---

Medical Men wishing to join the Society are requested to communicate with the *Honorary Secretary*, 9 Richmond Hill, Clifton. *The Annual Subscription is 10/6.*

---

*Extracts from Journal By-laws.*

4.—The *Journal* shall be delivered free to Subscribers and also to Members of the Society whose subscriptions are not in arrear.

6.—All Subscriptions for the *Journal* shall be paid in advance annually.

---

Communications intended for insertion in the *Journal*, Books for Review, &c., should be sent to the Editor, J. GREIG SMITH, M.A., F.R.S.E., 16 Victoria Square, Clifton.

Exchange Journals and communications referring to the delivery of the *Journal* should be sent to the Publication Secretary, L. M. GRIFFITHS, 9 Gordon Road, Clifton, to whom Subscriptions are to be paid *in advance.*

The Subscription to the *Journal* for 1884 is 5/-.

Authors requiring Reprints of their Papers should communicate with the Publisher.

### The following Periodicals are received in Exchange with the Bristol Medico-Chirurgical Journal.

AMERICA.

*Weekly.*

*The Medical Record.* New York: Wm. Wood & Co.

*The New York Medical Journal.* New York: D. Appleton & Co.

*Fortnightly.*

*Philadelphia Medical Times.* Philadelphia: J. B. Lippincott & Co.

*Monthly.*

*Index Medicus.* New York: F. Leypoldt.

*The Therapeutic Gazette.* Detroit: George S. Davis.

FRANCE.

*Three times a week.*

*Gazette des Hôpitaux.* Paris: 4 Rue de l'Odéon.

*L'Union Médicale.* Paris: 11 Rue de la Grange-Batelière.

GREAT BRITAIN.

*Weekly.*

*British Medical Journal.* London: 161A Strand.

*The Edinburgh Clinical and Pathological Journal.* Edinbugh: Bell & Bradfute.

*Monthly.*

*The Birmingham Medical Review,* Birmingham: Cornish Bros.

*The London Medical Record.* London: Smith, Elder, & Co.

*The Sanitary Record.* London: Smith, Elder, & Co.

*Edinburgh Medical Journal.* Edinburgh: Oliver & Boyd.

*The Glasgow Medical Journal.* Glasgow: Alex. Macdougall.

*Half-yearly.*

*The Liverpool Medico-Chirurgical Journal.* Liverpool: Medical Institution.

*The Retrospect of Medicine.* London: Simpkin, Marshall, & Co.

### *BOOKS RECEIVED.*

*Good Remedies—out of Fashion.* By Charles J. Hare, M.D. Cantab., F.R.C.P. London: J. & A. Churchill. 1883.

*Alcoholic Inebriety, from a Medical Standpoint.* By Joseph Parrish, M.D. Philadelphia: P. Blakiston, Son, & Co. 1883.

*Clinical Notes on Cancer.* By Herbert L. Snow, M.D. Lond., and Surgeon to the Cancer Hospital, Brompton. London: J. & A. Churchill. 1883.

*Asiatic Cholera.* A Report of an outbreak of Epidemic Cholera in 1876 at a Camp near Murree. By Chas. Moore Jessop, M.R.C.P., Brigade Surgeon H.M.'s British Forces. London: H. K. Lewis. 1883.

*A Manual of Midwifery for Midwives.* By Fancourt Barnes, M.D., M.R.C.P. Second Edition. London: Smith, Elder, & Co. 1883.

---

### BRISTOL MEDICO-CHIRURGICAL JOURNAL.

### TERMS FOR ADVERTISEMENTS.

| | | Special Places. | Outside of Cover. |
|---|---|---|---|
| | £ s. d. | £ s. d. | £ s. d. |
| Whole Page ... ... ... | 1 1 0 | 1 11 6 | 2 2 0 |
| Half Page ... . ... | 0 12 6 | 0 17 6 | 1 5 0 |
| Quarter Page ... ... | 0 7 6 | 0 10 0 | 0 15 0 |

Insets (single leaf), 5/-.

The next number of the *Journal* will be published in March, 1884.

Orders for Advertisements should be sent to the Publication Secretary, L. M. Griffiths, 9 Gordon Road, Clifton.

### THE "HANDY" SYSTEM

OF

### MEDICAL BOOK-KEEPING,

BY

ALFRED SHEEN, M.D.,

**Surgeon to the Glamorganshire and Monmouthshire Infirmary, Cardiff.**

ENTERED AT STATIONERS' HALL.

The usual plan of **DAY BOOK AND LEDGER** is possibly painfully familiar to most Medical Men. The principle of it seems to be, in two words, "accumulated labour." A man goes on from day to day recklessly tying knots in the string of his work, all of which, at the end of six or twelve months, he has to untie and re-arrange.

The "Handy" System is an attempt to simplify medical book-keeping, and it is claimed for it that a Medical Practitioner is able, if he will only take the trouble to learn the system—

1.—To reduce the labour of "posting" to a minimum.

2.—To keep book debts well in hand.

3.—To see, by an easy calculation, how much is booked year by year.

**THE DAY BOOK** is the centre of all clerical work, and may be used with any form of Ledger.

1.—The name and address, and other necessary particulars of each patient have only to be entered once during a month.

2.—Every necessary entry respecting each patient, except the prescription, is entered daily on one line running straight across the page.

3.—It contains an epitome of all the work and should be constantly at hand.

4.—The total amount to be entered in the Ledger, at the end of the month, is stated in the cash column provided for that purpose.

THE LEDGER is indexed so that the accounts can be entered under each client's name alphabetically.

It is presumed that accounts are sent out at least every six months. There are six columns for the six months, and to these columns the amounts in the day book are transferred at the end of each month. No particulars are entered in the Ledger; if these are wanted they can be readily got at by referring back to the Day Book. At the end of six months, or whenever the account is rendered, the total amount is entered in the cash column provided for that purpose. The next two columns show when the account is rendered and when paid, and the last cash column is for amounts brought forward where the previous account is not paid or only part paid. The clients as they are entered in the Ledger are numbered consecutively, and on a re-entry of any particular client he retains his original number. There is also a column for the charge per visit or consultation, which when once filled in is useful as a guide in future attendances. For complicated accounts a small ordinary Ledger had better be used.

PRESCRIPTION SLIPS may be used instead of a Prescription Book, and are much more handy. A sheet of foolscap will cut into eight of them. These, with the patient's name and address, age, &c., at the top, may contain the prescriptions on one side and notes of the case on the other. After one is used it should be numbered, and the next one for the same patient will be No. 2, and so on. These slips are kept together in a case or by an ordinary elastic band, on the Consulting Room table, arranged alphabetically. As they are done with they are taken out and placed in a drawer, arranged alphabetically; they are then at hand when wanted again for another attendance. These slips are a great advantage in many ways; for instance, if notes are taken on them, groups of similar cases may be arranged at any time for reference or study. They are a great incentive to case taking, an important element of a general practitioner's daily routine which, as a rule, is neglected.

The learning of this method of course involves a certain amount of trouble, but what that is worth anything does not? It is claimed for it that it reduces chaos to order, and is easily learnt and carried into practice.

---

### PRICES:

| | | |
|---|---|---|
| DAY BOOK, Foolscap Folio, or 13½ by 8¼ in. .. | 2½ Quires, 9/6; | 5 Quires, 14/6. |
| LEDGER, Large Post Quarto, or 11 by 9 in. .. | 2½ Quires, 8/6; | 5 Quires, 12/6. |

ALSO, BY THE SAME AUTHOR,

| | |
|---|---|
| THE WORKHOUSE AND ITS MEDICAL OFFICER .. .. .. | Price 2/-. |
| THE HANDY VISITING LIST, size 5⅝ by 2½ in. .. .. .. | 3/- per doz. |
| THE HANDY TEMPERATURE CHART, size 5⅝ by 2½ in. .. .. | 3/- per 100. |

---

*Published by WM. LEWIS, Bookseller, Cardiff.*

